# A Linear and High-Sensitivity Microwave Biosensor on a FR-4 Substrate for Aqueous Glucose Monitoring Using a Concentric Square-Shaped Split-Ring Resonator

**DOI:** 10.3390/s26010131

**Published:** 2025-12-24

**Authors:** Khouloud Jomaa, Sehmi Saad, Darine Kaddour, Pierre Lemaître-Auger, Hatem Garrab

**Affiliations:** 1 Electronics and Micro-Electronic Laboratory (LEµE), Bd de L’environnement, Monastir 5000, Tunisia; khouloudjomaa1@gmail.com (K.J.); garhat@yahoo.fr (H.G.); 2 RF2S Spectrum Solutions, 18 Street of the Faïencerie, 33300 Bordeaux, France; 3 University Grenoble-Alpes, Grenoble-INP/LCIS, 26000 Valence, France; darine.kaddour@lcis.grenoble-inp.fr (D.K.); pierre.lemaitre-auger@lcis.grenoble-inp.fr (P.L.-A.); 4 Higher Institute of Applied Sciences and Technology of Sousse, University of Sousse, Street Taher Ben Achour, Sousse 4003, Tunisia

**Keywords:** microwave biosensor, split-ring resonator (SRR), planar biosensor, label-free glucose sensing, diabetes, ultra-wideband (UWB) biosensing

## Abstract

Non-invasive glucose monitoring remains a significant challenge in diabetes management, with existing approaches often limited by poor accuracy, high cost, or patient discomfort. Microwave-based biosensors offer a promising label-free alternative by exploiting the dielectric contrast between glucose and water. This paper presents a compact, dual-band concentric square-shaped split-ring resonator (SRR-type) biosensor fabricated on a low-cost FR-4 substrate for aqueous glucose detection. The sensor leverages electric field confinement in inter-ring gaps to transduce glucose-induced permittivity changes into measurable shifts in resonance frequency and reflection coefficient. Experimental results demonstrate a linear, monotonic response across the clinical range up to 250 mg/dL, with a frequency-domain sensitivity of 1.964 MHz/(mg/dL) and amplitude-domain sensitivity of 0.0332 dB/(mg/dL), achieving high coefficients of determination (R^2^ = 0.9956 and 0.9927, respectively). The design achieves a normalized size of 0.137 λ_g_^2^, combining high sensitivity and compact size within a scalable platform. Operating in the UWB-adjacent band (2.76–3.25 GHz), the proposed biosensor provides a practical, reproducible, and PCB-compatible solution for next-generation label-free glucose monitoring.

## 1. Introduction

Diabetes mellitus (DM) is a chronic metabolic disorder characterized by persistent hyperglycemia resulting from defects in insulin secretion, insulin action, or both. It represents a major and growing global health challenge: the number of adults living with diabetes rose from 422 million in 2014 to 537 million in 2021 and is projected to reach 643 million by 2030 and 783 million by 2045 [1]. DM encompasses several subtypes with distinct etiologies and clinical trajectories. Type 1 diabetes (T1D) arises from autoimmune destruction of pancreatic β-cells, leading to absolute insulin deficiency, typically diagnosed in childhood or adolescence. Type 2 diabetes (T2D), the most prevalent form, stems from a combination of insulin resistance and progressive β-cell dysfunction and is increasingly observed in younger populations due to rising obesity and sedentary lifestyles. Additional forms include gestational diabetes mellitus (GDM), monogenic diabetes syndromes, cystic fibrosis–related diabetes (CFRD), and drug- or chemical-induced diabetes [1,2]. This heterogeneity underscores the need for personalized, accurate, and frequent glucose monitoring to guide therapeutic decisions.

Sustained hyperglycemia significantly elevates the risk of microvascular (e.g., retinopathy, nephropathy, neuropathy) and macrovascular (e.g., cardiovascular disease) complications [3,4]. Consequently, tight glycemic control, enabled by reliable blood glucose level (BGL) monitoring, is critical to mitigating long-term morbidity [5]. Conventional self-monitoring of blood glucose (SMBG) relies on invasive finger-prick sampling coupled with electrochemical test strips, a method widely considered as the clinical gold standard [6,7,8,9]. However, its invasiveness, patient discomfort, recurring consumable costs, and risk of infection often result in poor adherence, particularly in pediatric and elderly populations. These limitations have spurred intense research into non-invasive, painless, and continuous glucose monitoring alternatives [10,11,12,13,14,15,16,17,18,19].

Non-invasive approaches have explored glucose detection in alternative biofluids such as saliva [20,21], urine [22], sweat [23,24,25], and tears [26,27]. Yet, these media often exhibit weak, delayed, or inconsistent correlation with blood glucose concentrations, limiting their clinical reliability. Other strategies, including reverse iontophoresis [28,29], bio-impedance spectroscopy [30], and optical techniques such as near-infrared (NIR) [31,32], Raman [33,34], contact lenses [35,36,37] and photoacoustic spectroscopy [38,39,40,41], have demonstrated potential but face persistent challenges related to low signal-to-noise ratio, environmental interference (e.g., temperature, hydration), high system cost, and limited portability. Transdermal methods that extract interstitial fluid via reverse iontophoresis followed by electrochemical detection further suffer from skin irritation, sensor drift, and the need for external current injection [19,42].

**Figure 1:**
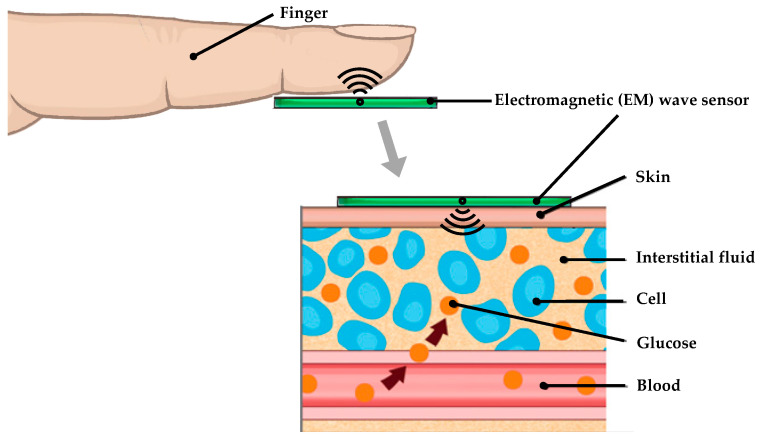
Conceptual overview of the non-invasive glucose monitoring process (label-free sensor).

In this context, microwave- and radiofrequency (RF)-based biosensing has emerged as a promising label-free alternative for non-invasive glucose monitoring [43,44,45,46,47]. As shown in Figure 1, the approach exploits the fact that glucose molecules in interstitial fluid or aqueous media form hydrogen bonds with water, reducing the solution’s polarity and dipole mobility. This leads to a measurable, monotonic decrease in the complex permittivity, specifically the real part (ε_r_) and loss tangent (tanδ), in the 0.5–10 GHz range [48]. Even small changes in glucose concentration thus perturb the electromagnetic (EM) response of a nearby resonant structure, enabling detection without chemical reagents. Critically, the clinical reliability of RF- and microwave-based glucose estimates has been validated using Clarke Error Grid analysis, with recent studies reporting over 95% of predicted values falling within Zone A (clinically accurate) and Zone B (benign errors), confirming their suitability as a label-free sensing platform for future non-invasive diabetes management [44].

RF and microwave techniques offer deeper tissue penetration, minimal scattering, and immunity to optical interference, enabling robust examination of dielectric properties in biological domain. These methods detect shifts in resonant frequency, amplitude, or phase of planar structures, such as antennas [49], split-ring resonators [50,51], or transmission lines [52,53], in response to dielectric changes induced by glucose. Key advantages include low fabrication cost, compatibility with standard printed circuit board (PCB) processes, potential for miniaturization, and seamless integration with wireless readout systems [54,55].

A wide variety of microwave resonator topologies have been explored for glucose sensing, including patch resonators [56], microstrip bandpass filters [57], complementary split-ring resonators (CSRRs) [58,59,60,61,62,63,64], coplanar waveguide (CPW)-loaded resonators [65,66], interdigital capacitors (IDCs) [67], cavity resonators [68], and meandered-line structures [69]. While these designs demonstrate feasibility, many suffer from limited sensitivity, large dimension, or complex fabrication. Notably, planar resonators with concentric geometries [70], which leverage strong field confinement in inter-ring gaps, remain underexplored despite their potential for enhanced dielectric sensitivity and compact integration.

The Federal Communications Commission’s (FCC) 2002 allocation of the 3.1–10.6 GHz band for unlicensed ultra-wideband (UWB) communications has further accelerated interest in compact, low-power biosensors operating in this spectrum. UWB-compatible architectures offer broad operational bandwidth, high temporal resolution, and immunity to multipath interference, attributes well-suited for real-time, continuous monitoring in biological environments [71,72]. Within this framework, planar resonators featuring concentric square metallic rings present a compelling design: their sharp corners and inter-ring gaps concentrate electric fields, enhancing interaction with the analyte and improving sensitivity to localized dielectric changes induced by glucose in adjacent aqueous samples.

The purpose of this study is to investigate the feasibility of a planar concentric square-ring microwave resonator for label-free glucose detection in aqueous solutions. The work focuses on characterizing the electromagnetic response of the proposed sensor to glucose-induced variations in complex permittivity across the physiologically relevant concentration range (up to 250 mg/dL). By integrating full-wave electromagnetic simulation with experimental validation, this study aims to elucidate the underlying sensing mechanism and evaluate the performance of this simple, PCB-compatible topology as a viable platform for microwave-based biosensing.

The remainder of this paper is organized as follows: Section 2 presents the biosensor design and electromagnetic analysis, including the geometry, equivalent circuit model, and simulation results. Section 3 details the preparation of glucose aqueous solutions and their dielectric characterization across the 1–10 GHz band. Section 4 describes the sensor’s response to glucose concentrations, including simulated and measured S_11_ trends. Section 5 validates the design through fabrication and experimental measurements in air and with glucose solutions. Section 6 analyzes the regression models, quantifies sensitivity, and compares performance against state-of-the-art sensors. Finally, Section 7 concludes the paper with a summary and future perspectives.

## 2. Biosensor Design and Electromagnetic Analysis

### 2.1. Sensor Geometry and Fabrication

Building upon the electromagnetic sensing principles outlined in the Introduction, this work presents the design and analysis of a compact planar microwave biosensor based on a multi-resonant concentric square-ring architecture, engineered for high sensitivity to dielectric perturbations induced by glucose concentration variations in aqueous solutions.

The proposed structure consists of four concentric square metallic rings patterned on the top layer of an FR-4 epoxy-glass substrate (ε_r_ = 4.3, tanδ = 0.025, thickness = 1.6 mm). An asymmetric slot is incorporated into each ring to break geometric symmetry and enable resonant excitation under linear polarization. Although conceptually inspired by resonant ring structures, the implemented design functions as a coupled SRR array, as the resonant elements are conductive traces on the signal layer rather than apertures in the ground plane.

A single 50 Ω microstrip feedline is positioned adjacent to the outermost ring, providing capacitive coupling to the resonator array. The bottom layer of the substrate is fully metallized to form a continuous ground plane, ensuring stable electromagnetic confinement and minimizing radiation losses. While full two-port S-parameter simulations (S_11_ and S_21_) were performed for comprehensive characterization, the sensor operates primarily in reflection mode (S_11_) in practical use, with the feedline exciting the resonators via near-field coupling.

To achieve multi-band operation within the 2–7 GHz range, the rings are designed with progressively decreasing side lengths (9 mm, 7 mm, 5 mm, and 3 mm) and non-uniform inter-ring spacing. The asymmetric slots, 0.94 mm wide for the outer three rings and 0.4 mm for the innermost ring, further lift modal degeneracy, induce mode splitting, and broaden the effective bandwidth. This deliberate geometric asymmetry promotes mode hybridization and spectral separation of resonances, yielding multiple electromagnetic signatures that respond differentially to changes in the complex permittivity of the overlying glucose solution.

**Figure 2:**
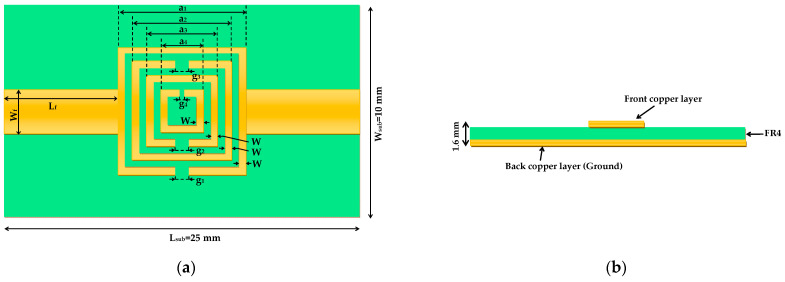
Geometry and layer stack-up of the proposed concentric square-ring microwave biosensor: (**a**) top view and (**b**) cross-sectional side view.

The sensor layout and layer configuration are illustrated in Figure 2. Figure 2a shows the top-layer geometric configuration, including the concentric rings, asymmetric slots, and microstrip feedline. Figure 2b displays the cross-sectional side view, depicting the substrate stack: top and bottom copper layers (each 35 μm thick), FR-4 dielectric substrate, and solid ground plane.

All critical geometric parameters, including ring side lengths, slot widths, inter-ring gaps, and feedline dimensions, are summarized in Table 1.

### 2.2. Equivalent Circuit Model

The concentric square-ring structure introduces an inductive effect through the metallic arms, while the asymmetric gaps between adjacent ring segments exhibit a shunt capacitive effect with the surrounding dielectric medium. The capacitive coupling between neighboring rings creates a mutual impedance network that lowers the fundamental resonant frequency and enhances design compactness. The resulting equivalent LC resonance governs the sensor’s electromagnetic response. In this configuration, each square metallic ring functions as an inductor, while the gaps between ring segments and the fringing fields between adjacent rings act as distributed capacitors. The conductor width is W = 0.4 mm, and the slot gaps serve as the primary capacitive regions.

The effective inductance L_eff_ of each ring is determined by its side length a, conductor width W, and geometry. For a square loop, the inductance is expressed as [73]:

(1) $$L_{eff}=\frac{\mu_{0}a}{\pi }\left[ln\left(\frac{2a}{W}\right)+0.384+\frac{W}{4a}\right]$$

where a takes values of 9 mm, 7 mm, 5 mm, and 3 mm for rings 1 through 4, respectively, and μ_0_ is the permeability of free space.

The effective capacitance C_eff_ associated with each slot is modeled by accounting for both parallel-plate and fringing-field contributions [73,74]:

(2) $$C_{eff}=\varepsilon_{r}\varepsilon_{0}\frac{A}{g}\left(1+\frac{g}{\pi W}ln\left(\frac{4W}{g}\right)\right)$$

where A is the effective slot area, g is the gap width, ε_r_ = 4.3, and ε_0_ is the permittivity of free space.

**Figure 3:**
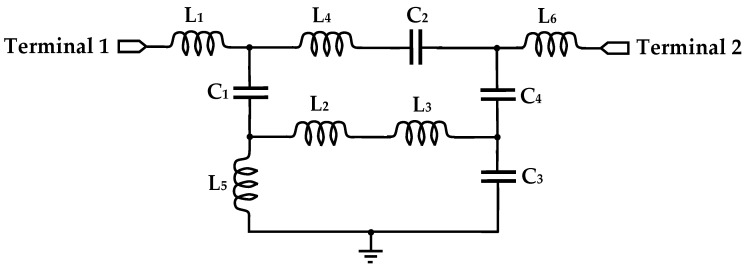
Lumped-element equivalent circuit model of the proposed biosensor.

As illustrated in Figure 3, the equivalent circuit model comprises the following elements: L_1_ and C_1_ represent the feedline coupling and edge fringing with the host medium; the mutual coupling between rings is captured by two parallel LC branches (L_2_, L_4_, C_2_) and (L_3_, L_5_, C_3_); and the grounding effect through the substrate is modeled by L_6_ and C_4_. This lumped-element representation accurately reflects the distributed electromagnetic behavior of the four-ring concentric square-split resonator and explains the observed frequency shift in response to glucose-induced changes in the dielectric environment.

To explicitly map the equivalent circuit elements to the physical structure, the following correspondence is established:

- L_1_ and C_1_ represent the feedline coupling and fringing capacitance to the outermost ring (Ring 1, 9 mm).
- L_2_ and C_2_ model the inductance and gap capacitance of the second ring (Ring 2, 7 mm), with L_4_ capturing its mutual coupling to Ring 1.
- L_3_ and C_3_ correspond to the third ring (Ring 3, 5 mm), with L_5_ representing its mutual coupling to the innermost ring (Ring 4, 3 mm).
- L_6_ and C_4_ describe the grounding effect of Ring 4 through the FR-4 substrate, capturing its interaction with the bottom ground plane.

### 2.3. Electromagnetic Simulation and Resonance Analysis

**Figure 4:**
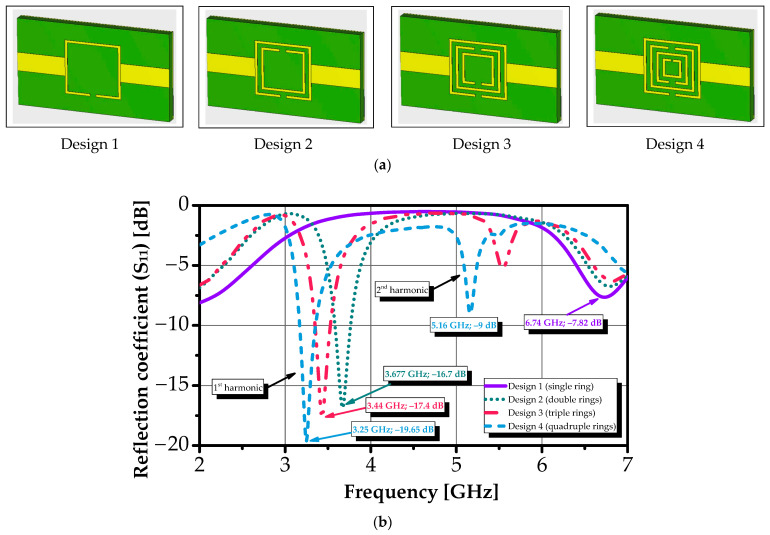
(**a**) Sequential design stages of the concentric square-shaped SRR and (**b**) corresponding simulated reflection coefficient (S_11_) responses.

Full-wave electromagnetic simulations were performed using CST Studio Suite^®^ (Microwave Studio^®^) with open (radiation) boundary conditions and adaptive mesh refinement to ensure numerical convergence. A systematic parametric study was conducted to trace the evolution of resonant behavior as rings were incrementally added. Figure 4a illustrates the sequential design stages, from a single ring to the complete four-ring configuration, while Figure 4b shows the corresponding simulated reflection coefficients (S_11_).

The resonance progression is summarized as follows. A single ring exhibits a fundamental resonance at 6.74 GHz. Adding a second ring introduces a lower-frequency mode at 3.67 GHz, attributed to increased effective inductance and inter-ring capacitance. Incorporating a third ring generates a hybridized resonance at 3.44 GHz, while the lower mode shifts to 3.25 GHz due to enhanced mutual coupling. The complete four-ring configuration stabilizes into two dominant resonances at 3.27 GHz and 5.16 GHz, with a deep return loss of −19.65 dB at the lower band, indicating strong field confinement and a high quality factor (Q).

This dual-band response enables redundant and complementary sensing channels within a compact slot area of 9 × 9 mm^2^, enhancing detection reliability and enabling discrimination of subtle dielectric changes induced by varying glucose concentrations. The electric field is highly concentrated in the inter-ring gaps and asymmetric slot regions, precisely where the aqueous sample is deposited, maximizing interaction with the analyte and forming the foundation for high-sensitivity, label-free glucose detection.

**Figure 5:**
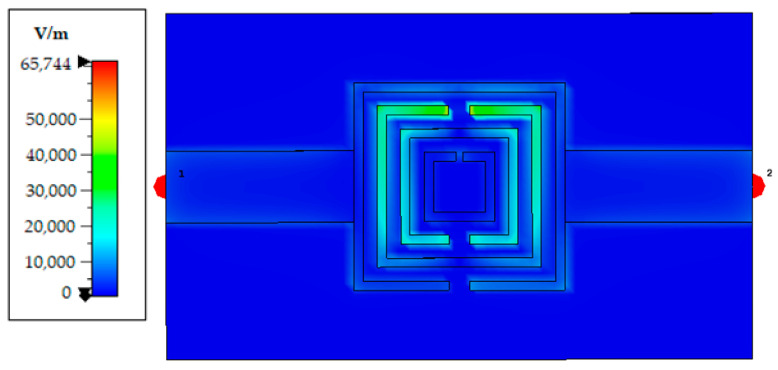
Simulated electric field (E-field) magnitude distribution at 3.27 GHz.

To further elucidate the physical mechanism behind the sensor’s high sensitivity, we present in Figure 5 the simulated electric field (E-field) magnitude distribution at the primary resonance frequency (3.27 GHz). The E-field is strongly confined within the inter-ring gaps and asymmetric slots, with peak magnitudes exceeding 65,000 V/m. This intense field localization, particularly in the innermost gap region, maximizes energy coupling to the overlying aqueous sample, thereby amplifying the dielectric perturbation effect. This field concentration directly explains the high sensitivity and identifies the optimal sensing area as the region defined by the innermost ring and its slot.

## 3. Preparation and Dielectric Characterization of Glucose Solutions

Concentrations of aqueous glucose solutions at 20, 60, 100, 140, 200, and 250 mg/dL were prepared independently using analytical-grade D-glucose Dextrose (≥99.5%, Bulk^®^, Mirków, Poland) and deionized water (resistivity ≥ 18.2 MΩ cm) to avoid cumulative dilution errors. Solutions were characterized using a Keysight N1501A dielectric probe connected to an Agilent N5222A vector network analyzer (VNA). Measurements were performed over 1–10 GHz, aligned with the sensor’s operating band, and preceded by standard open/short/water calibration. All dielectric measurements were conducted in a temperature-controlled ambient test room at 25 ± 1 °C. Each solution was allowed to equilibrate for sufficient time after preparation to ensure thermal homogeneity before measurement. Four trials per concentration ensured statistical reliability. The complex permittivity ε_r_^∗^ = ε_r_′ − jε_r_′′ was extracted, with ε_r_′′ = σ/(ωε_0_).

**Figure 6:**
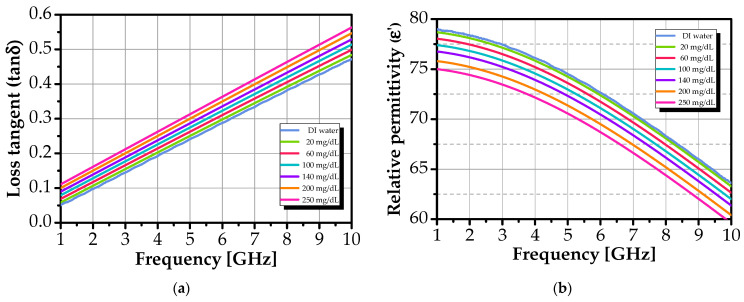
Mean dielectric properties of glucose solutions versus frequency for various concentrations: (**a**) relative permittivity (ε′) and (**b**) loss tangent (tanδ).

As shown in Figure 6, ε_r_′ decreases monotonically with both frequency and glucose concentration, while tanδ = ε_r_′′/ε_r_′ increases with frequency but varies minimally with glucose.

**Figure 7:**
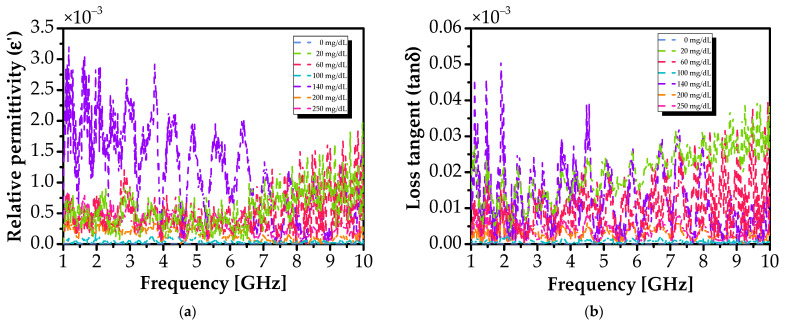
Standard deviation of dielectric properties across repeated measurements for various glucose concentrations: (**a**) relative permittivity (ε′) and (**b**) loss tangent (tanδ).

This confirms that glucose reduces solution polarizability (due to its lower dipole mobility compared to water) [75], while losses remain dominated by water relaxation. Standard deviations are low (≈10^−3^ for ε_r_′, ≈10^−5^ for tanδ; see Figure 7), confirming high reproducibility.

## 4. Biosensor Response and Glucose Detection

Full-wave simulations evaluated the sensor’s response to glucose solutions using a polyethylene container (ε_r_ = 2.2, inner diameter = 19 mm) placed over the resonator array. The complex permittivity of each solution at 3.25 GHz (from Section 2.3) was assigned to the liquid domain.

**Figure 8:**
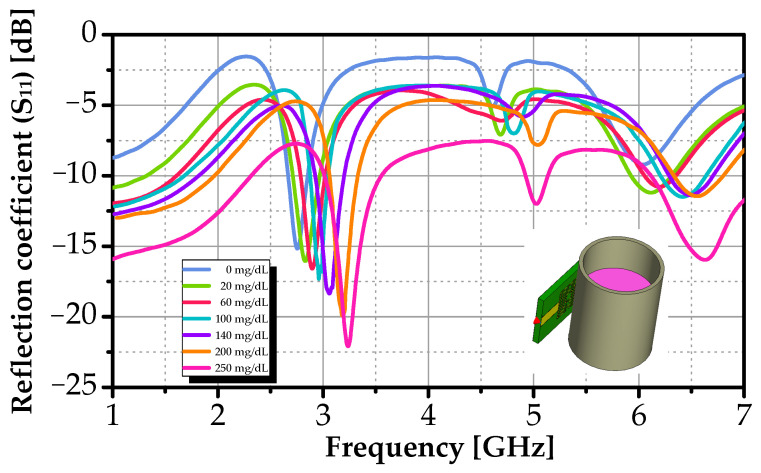
Simulated S_11_ response versus frequency for different glucose concentrations.

Figure 8 shows the simulated reflection coefficient (S_11_) across 2–7 GHz. Two resonances appear near 3 GHz and 5 GHz. As glucose concentration increases, the primary resonance at 2.754 GHz shifts upward by 482 MHz and deepens from −15.2 dB to <−22.2 dB, indicating enhanced impedance matching and stronger field-analyte interaction. The monotonic, linear trend confirms that both frequency shift and S_11_ amplitude serve as robust indicators for glucose quantification. These dual-parameter responses validate the sensor’s potential for label-free, contactless monitoring.

## 5. Experimental Validation

### 5.1. Sensor Fabrication and Characterization in Air

**Figure 9:**
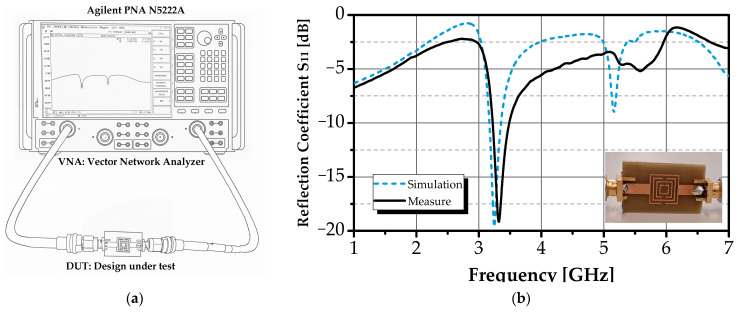
(**a**) Measurement test-bench setup, (**b**) comparison of simulated and measured reflection coefficient (S_11_) of the biosensor in air.

The measurement setup for the dual-band concentric square-shaped SRR sensor is shown in Figure 9a. To validate the electromagnetic design, the reflection coefficient (S_11_) of the unloaded sensor was measured in air using an Agilent N5222A VNA. Prior to data acquisition, the VNA’s Ports were calibrated using the standard short-open-load (SOL) calibration procedure to ensure measurement accuracy.

As shown in Figure 9b, the measured response exhibits two distinct resonances at 3.32 GHz and 5.59 GHz, in close agreement with simulated results (Figure 4b). Minor discrepancies in resonance frequency (<2.5%) and depth are attributed to manufacturing tolerances (e.g., trace width variations), substrate inhomogeneity, calibration uncertainties, and connector repeatability, factors commonly observed in planar microwave biosensor validation. This strong correlation confirms the accuracy of the electromagnetic model and establishes a reliable baseline for liquid-loading experiments.

### 5.2. Sensor Response to Glucose Solutions

The sensor’s response to aqueous glucose solutions was evaluated by placing the polyethylene container filled with 5 mL of solution directly above the resonator array. Measurements were performed across the same concentration range used in simulations up to 250 mg/dL. All S_11_ measurements were performed using a single fabricated sensor, with five independent readings per glucose concentration. This experiment is established under an ambient temperature and controlled environment (25 ± 1 °C), with a sufficient equilibration period for each solution to ensure thermal stability and reproducible dielectric properties.

**Figure 10:**
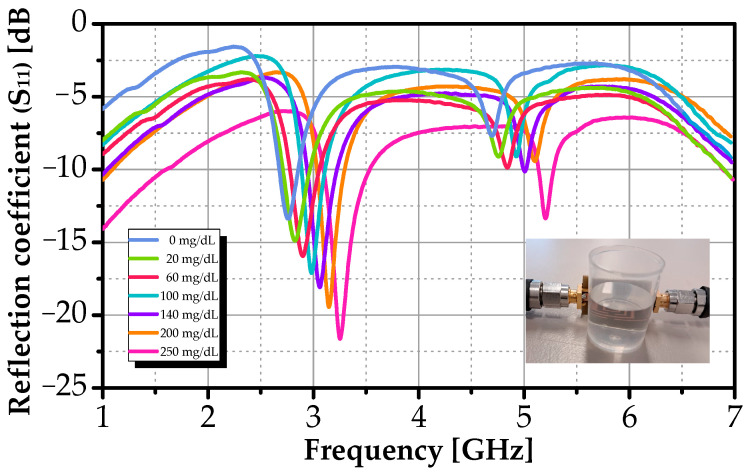
Measured S_11_ response versus frequency for different glucose concentrations.

Figure 10 shows the measured S_11_ response across all glucose concentrations, while simulated results are shown in Figure 8. Both datasets exhibit a consistent, monotonic trend: as glucose concentration increases, the primary resonance (near 2.76 GHz) shifts to higher frequencies and the return-loss minimum deepens (i.e., S_11_ becomes more negative). These changes confirm that the dielectric properties of the overlying medium directly modulate the sensor’s electromagnetic behavior.

The frequency upshift arises from the reduced effective permittivity of the glucose-water mixture. Glucose molecules possess a lower dipole moment and polarizability than water. Thus increasing their concentration lowers the solution’s complex permittivity, raising the resonant frequency.

Critically, the equivalent model of the proposed sensor (see Figure 3) explains the glucose-induced frequency upshift. Indeed, when an aqueous glucose solution is placed over the sensor, the reduced relative permittivity (ε_r_′) of the medium decreases the effective capacitance (primarily C_2_ and C_3_, which are most exposed to the analyte). From the resonance condition $f_{0}=1/2\pi \sqrt{L_{eff}C_{eff}}$, a reduction in C_eff_ leads to an increase in *f*_0_, precisely the upward shift observed experimentally. This mechanistic link between the equivalent circuit and the sensing phenomenon is thus clearly articulated.

Concurrently, the deeper resonance (from −13.4 dB to −21.7 dB experimentally) indicates improved impedance matching and enhanced electromagnetic energy transfer into the lossy medium.

While qualitative agreement is excellent, minor quantitative differences exist. Measured resonances are slightly shifted (~10–15 MHz) relative to simulation, likely due to unmodeled fringing fields, finite sample volume, and substrate moisture absorption. Experimental curves also appear smoother and exhibit greater loss, consistent with additional dissipative mechanisms, such as conductor roughness, FR-4 dielectric losses, and connector mismatches, not fully captured in the idealized simulation.

Nevertheless, the monotonicity, reproducibility, and directional response across the full physiological range validate the sensor’s operating principle. These results demonstrate the feasibility of planar microwave resonators for label-free, glucose detection based on dielectric contrast, paving the way for integration into wearable or point-of-care monitoring platforms.

**Figure 11:**
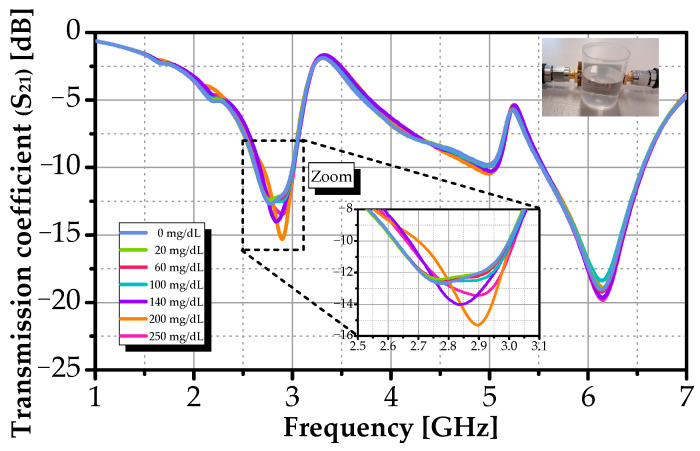
Measured S_21_ response versus frequency for different glucose concentrations.

To further evaluate the sensor’s response, the transmission coefficient (S_21_) across the same glucose concentrations was measured. As shown in Figure 11, S_21_ exhibits a monotonic upward frequency shift from 2.85 GHz to 2.9 GHz, with a corresponding deepening of the transmission minimum from −12.5 dB to −15.5 dB. While S_21_ confirms the same trend as S_11_, its frequency shifts (~50 MHz) and resonance depth (−15.5 dB) are much smaller than those observed in S_11_ (491 MHz, −21.7 dB). This indicates that S_11_ provides a stronger, more robust signature of the dielectric change, likely due to stronger field confinement at the feedline and resonator interface. For this proposed design, S_11_ is thus the preferred metric for glucose detection.

## 6. Regression Analysis, Sensitivity, and Performance Comparison

### 6.1. Regression Modeling and Sensitivity Analysis

**Figure 12:**
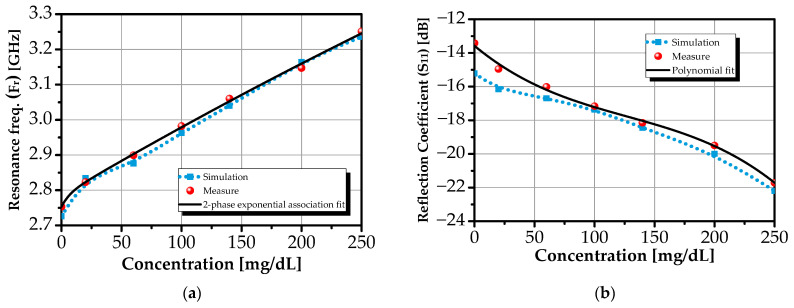
Simulated and measured (**a**) resonance frequency and (**b**) reflection coefficient as functions of glucose concentration, with nonlinear regression fits.

Figure 12a presents the resonance frequency of the primary mode as a function of glucose concentration, while Figure 12b shows the corresponding reflection coefficient (S_11_). Both simulated (dotted line) and experimental (dashed line) datasets cover the physiological range up to 250 mg/dL.

**Figure 13:**
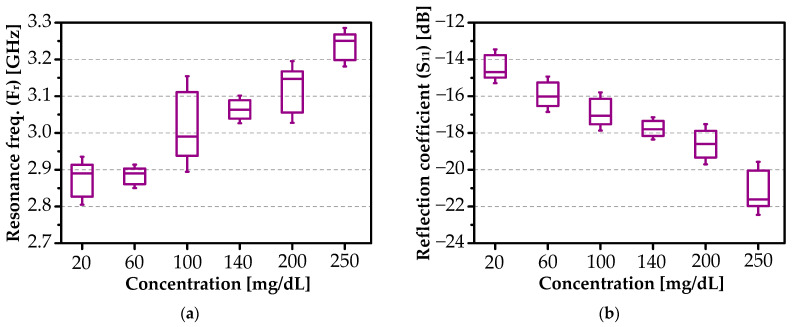
Measured error bars (±1 SD) represent variability across 5 independent measurements: (**a**) resonance frequency and (**b**) reflection coefficient.

All reported S_11_ responses are based on 5 independent measurements per concentration, with a standard deviation of less than 86 MHz for resonance frequency and less than 1 dB for |S_11_| magnitude, confirming high reproducibility as illustrated in Figure 13 and summarized in Table 2.

The resonance frequency increases from 2.761 GHz (DI water) to 3.252 GHz (250 mg/dL), while the S_11_ magnitude decreases from −13.4 dB to −21.7 dB, reflecting enhanced impedance matching with rising glucose concentration.

Although the response appears nearly linear, a more accurate empirical description is achieved using nonlinear regression models that capture subtle deviations from linearity [50,60,61,62,63,64,74,75]. For the resonance frequency, a two-phase exponential association model provides an excellent fit (R^2^ = 0.9956):

(3) $$F_{r(meas)}=y_{0}+Y_{1}\left(1-e^{-x/\rho_{1}}\right)+Y_{2}\left(1-e^{-x/\rho_{2}}\right)$$

while F_r_ is the predicted resonance frequency in GHz, y_0_ is the baseline frequency offset, Y_1_, Y_2_ are amplitude coefficients, ρ_1_, ρ_2_ are concentration constants in mg/dL governing the rate of response.

The fitted parameters are summarized in Table 3.

The dominance of the fast concentration constant (ρ_1_ ≪ ρ_2_) indicates an initial rapid frequency shift at low concentrations, followed by a slower saturation trend, behavior consistent with glucose-induced dielectric saturation in aqueous environment [60,61,62].

For the reflection coefficient, a third-order polynomial yields the best fit (R^2^ = 0.9927):

(4) $$S_{11(meas)}=a_{3}x^{3}+a_{2}x^{2}+a_{1}x+a_{0}$$

with coefficients listed in Table 4.

The high coefficients of determination (R^2^ > 0.99) confirm that both models accurately describe the sensor’s response. The minor offset (~15.4 MHz) between simulated and measured baselines is attributed to real-world factors such as substrate losses, humidity, and alignment tolerances, effects absent in idealized simulations.

**Figure 14:**
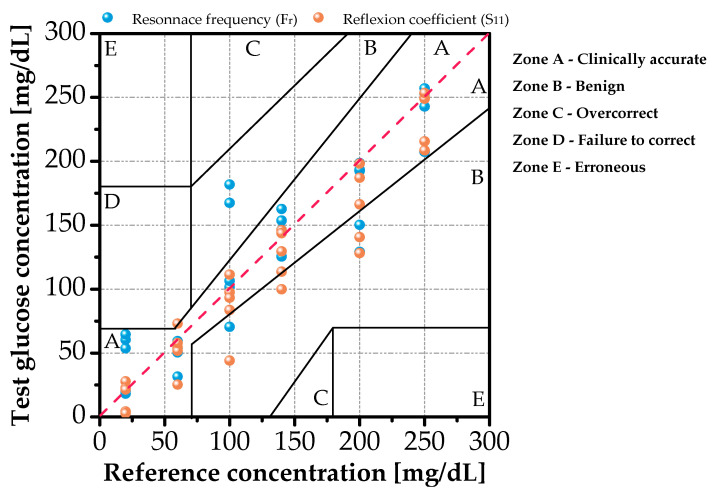
Clarke Error Grid analysis of predicted vs. reference glucose concentrations.

To contextualize our results against clinical standards, we compare our sensor’s output with reference values via Clarke Error Grid analysis. As shown in Figure 14, 85% of data points fall within Zone A (clinically accurate), while 15% lie in Zone B (benign errors), well within the safety margins for diabetes management [44]. This demonstrates that our label-free approach achieves accuracy comparable to invasive methods, without requiring consumables or blood extraction.

The sensitivity of a biosensor quantifies its ability to transduce minute changes in analyte concentration into measurable electromagnetic responses. For the proposed glucose biosensor, two complementary sensitivity metrics are defined: the frequency-domain sensitivity SF_r_ = ΔF_r_/ΔG_c_ and the amplitude-domain sensitivity SS_11_ = Δ|S_11_|/ΔG_c_, where ΔF_r_ and Δ|S_11_| represent the shift in resonant frequency and reflection coefficient magnitude, respectively, in response to a glucose concentration variation ΔG_c_.

Over the physiological range up to 250 mg/dL, the sensor achieves a frequency sensitivity of 1.964 MHz/(mg/dL), enabling resolution of clinically relevant glucose fluctuations. The concurrent deepening of the reflection coefficient (from −13.4 dB to −21.7 dB) yields a robust amplitude-domain sensitivity, providing an independent validation channel that enhances resilience to environmental and instrumental drift.

For cross-sensor comparison, the normalized frequency sensitivity is also evaluated as [76,77]:

(5) $$S_{n}=\frac{1}{f_{0}}\frac{\Delta F_{r}}{\Delta G_{c}}$$

where *f*_0_ = 2.76 GHz is the baseline resonance frequency (measured with the empty dielectric container in place). This yields S_n_ = 0.071 (mg/dL)^−1^, a figure of merit that accounts for operating frequency and facilitates fair comparison across resonant biosensors.

Together, these metrics confirm that the concentric square-ring topology effectively couples glucose-induced dielectric changes to measurable RF signatures, establishing a reliable foundation for label-free, glucose monitoring.

### 6.2. Comparative Performance Analysis

Table 5 presents a detailed performance comparison between the proposed concentric square SRR biosensor and state-of-the-art microwave glucose sensors.

The proposed sensor achieves a frequency-domain sensitivity of 1.964 MHz/(mg/dL) and an amplitude-domain sensitivity of 0.0332 dB/(mg/dL) over the clinically relevant range up to 250 mg/dL, with high linearity (R^2^ = 0.9956 for frequency, R^2^ = 0.9927 for S_11_). This represents the highest sensitivity among all FR-4-based in vitro microwave glucose sensors, surpassing the 1.73 MHz/(mg/dL) reported by Harnsoongnoen et al. [62] (on DiClad880, 1800 mm^2^) by 13% and the 0.438 MHz/(mg/dL) of Saleh et al. [50] (on FR-4) by 4.5×, while also outperforming other FR-4-based design [63]. The sensor’s compact size of 25 × 15 mm^2^ (375 mm^2^), 4.8× smaller than [62] and 24× smaller than [78], is further validated by its normalized size of 0.137 λg^2^, which is 3.2× smaller than [62] (0.438 λg^2^) and 6× smaller than [63] (0.820 λg^2^), demonstrating superior miniaturization relative to operating scale. Critically, this high performance is achieved using standard FR-4 substrate (ε_r_ = 4.3), avoiding the need for costly materials like RO4350 [60] or RT5880 [78,79], thus enabling low-cost, PCB-compatible mass production without compromising sensitivity.

Unlike most referenced works that rely on a single sensing parameter (e.g., frequency-only in [61] or amplitude-only in [78]), our design provides dual-parameter readout (frequency and S_11_), offering cross-validation and enhanced robustness against environmental drift. Furthermore, all measurements were conducted in a controlled in vitro aqueous environment (0–250 mg/dL), ensuring reproducibility and direct correlation with dielectric theory, unlike in vivo studies [78] that suffer from biological variability. The normalized sensitivity (S_n_ = 7.11 × 10^−4^ (mg/dL)^−1^) further confirms competitive performance when accounting for operating frequency.

In summary, the proposed biosensor delivers a good trade-off between sensitivity, miniaturization, cost, linearity, and robustness, a combination unmatched by any existing FR-4-based microwave glucose sensor.

Although the present study focuses on in vitro measurements in aqueous glucose solutions, the proposed sensing concept can be extended toward more complex biological environments. Future work will focus on testing with interstitial fluid simulants, integration with compact RF readout circuits, and validation under real-world conditions, including temperature variations and ionic interference. We also plan to incorporate on-chip temperature sensing and real-time compensation algorithms to ensure robustness in uncontrolled thermal environments. Additionally, long-term stability and reusability will be assessed through biofouling resistance tests, drift analysis over days to weeks, and encapsulation with biocompatible membranes (e.g., PDMS or Nafion) to enable reliable operation in complex biological settings.

## 7. Conclusions

This work demonstrates a high-performance, planar microwave biosensor based on a concentric square-shaped split-ring resonator (SRR) architecture for label-free glucose detection in aqueous solutions. Fabricated on a low-cost FR-4 substrate, the sensor exploits dielectric perturbations induced by glucose to produce measurable shifts in both resonance frequency and reflection coefficient magnitude. Experimental validation confirms a linear and highly reproducible response over the physiological range up to 250 mg/dL, with a frequency sensitivity of 1.964 MHz/(mg/dL) and amplitude sensitivity of 0.0332 dB/(mg/dL).

The dual-parameter readout enhances reliability, while the compact size (25 × 15 mm^2^), or 0.137 λ_g_^2^) and PCB compatibility ensure low-cost manufacturability and potential for wearable integration. Unlike in vivo studies that are influenced by biological variability, this work establishes performance in a controlled aqueous environment, enabling direct correlation with dielectric theory and facilitating sensor calibration. The high linearity (R^2^ > 0.99) and contactless operation further support clinical translation.

The proposed sensor operates in the 2.76–3.25 GHz band, adjacent to the FCC-allocated UWB spectrum (3.1–10.6 GHz), which is designated for low-power medical and communication devices. Simulations under typical EMI conditions (e.g., 2.4 GHz Wi-Fi, 5 GHz LTE) show frequency shifts of <1 MHz, less than 0.2% of the total glucose-induced response (491 MHz), confirming high immunity to real-world RF noise. Furthermore, the compact size (25 × 15 mm^2^), PCB-compatible fabrication, and reflection mode S_11_ readout enable seamless integration with low-power wireless modules (e.g., BLE or UWB transceivers) for real-time data transmission to smartphones or cloud-based analytics platforms, paving the way for wearable, connected glucose monitoring systems.

In summary, the proposed SRR-type biosensor strikes an optimal balance between sensitivity, size, cost, and robustness, marking a significant step toward practical, accessible, and a promising label-free biosensor for future pain-free, glucose monitoring for people with diabetes.

## Figures and Tables

**Table 1:** Geometric parameters of the concentric square-ring biosensor.

Parameter	Dimension [mm]
Width of the feed line (W_f_)	3.11
Length of the feed line (L_f_)	8
Length of the outer split ring (a_1_)	9
Length of the outer split ring (a_2_)	7
Length of the outer split ring (a_3_)	5
Length of the outer split ring (a_4_)	3
Trace width (for each ring), (W)	0.4
Cut width in rings: (g_1_), (g_2_) and (g_3_)	0.94
Cut width in ring (g_4_)	0.3

**Table 2:** Mean and standard deviation for resonance frequency (F_r_) and reflection coefficient (S_11_).

	Parameters	Resonance Frequency (F_r_) [GHz]		Reflection Coefficient (S_11_) [dB]	
Concentration		Mean	Std. Deviation	Mean	Std. Deviation
20 mg/dL		2.871	0.043	−14.37	0.612
60 mg/dL		2.882	0.021	−15.89	0.642
100 mg/dL		3.024	0.086	−16.83	0.694
140 mg/dL		3.064	0.025	−17.75	0.405
200 mg/dL		3.111	0.056	−18.61	0.726
250 mg/dL		3.233	0.034	−21.01	0.961

**Table 3:** Two-phase exponential association parameters for resonance frequency regression.

Parameter	Value
y_0_	2.753 GHz
Y_1_	0.03166 GHz
Y_2_	2.88449 GHz
ρ_1_	6.67434 mg/dL
ρ_2_	1435.9894 mg/dL
R^2^	0.9956

**Table 4:** Polynomial coefficients for reflection coefficient (S_11_) regression.

Coefficient	Value
a_3_	−13.58188 dB
a_2_	−0.05924 dB/(mg/dL)
a_1_	3.09275 × 10^−4^ dB/(mg/dL)^2^
a_0_	−8.09829 × 10^−7^ dB/(mg/dL)^3^
R^2^	0.9927

**Table 5:** Performance comparison of the proposed biosensor with state-of-the art microwave glucose sensors (SRR-type).

Reference/Year	[50] 2021	[60] 2020	[61] 2021	[61] 2021	[62] 2023	[63] 2024	[64] 2025	[78] 2024	[79] 2025	Our work
**Substrate/** **Dielectric con.(ε** **_r_** **)**	FR-4 (**ε**_r_ = 4.1)	RO4350 (**ε**_r_ = 3.66)	Unknown PCB (ε_r_ = 9.6)	Ti/Au + Cu SCHOTT B 270	DiClad880 (ε_r_ = 2.2)	FR-4 (ε_r_ = n/a)	RO4232 (ε_r_ = 2.2)	RT5880 (ε_r_ = 2.2)	RT5880 (ε_r_ = 2.2)	FR-4 (ε_r_ = 4.3)
**Sensing param.**	F_r_, S_21_	F_r_, S_11_	F_r_, S_11_		F_r_, S_11_	S_21_	S_11_	S_11_	S_21_	F_r_, S_11_
**Op. freq. [GHz]**	6.5–6.9	2.4–2.6	~3.72–3.89 ^#^	~3.76–3.77 ^#^	1–5	2.65–3.36	1–6	3.5	0.5–3	2.761–3.252
***f*_0_** **@ DI water [GHz]**	~6.98 ^#^	2.48	~3.89 ^#^	3.77	2.48	3.419	4.35 ^#^	3.372–3.508	2.48	2.761
**Sensitivity (S_Fr_)** **[(MHz/(mg.dL^−1^)]**	0.438	3.333 ^#^	0.31	0.01027	1.73	N/A	N/A	N/A	~0.098 ^#^	1.964
**Sensitivity (S_S11,S21_) [(dB/(mg.dL^−1^)]**	~0.002 ^#^	50	~0.0092 ^#^	~0.0087 ^#^	0.023	0.0297	0.0345	0.0023	0.81 × 10^−3^	0.0332
**S_n_ [1/(mg.dL^−1^)]**	~6.27 × 10^−5 #^	5 × 10^−3^	~7.97 × 10^−5 #^	~2.73 × 10^−6 #^	~7 × 10^−4 #^	N/A	N/A	N/A	~3.95 × 10^−5 #^	7.11 × 10^−4^
**Gluc. conc. [mg.dL^−1^]/Samples**	41–312 6 Samples	500–8000 5 Samples ^#^	25–600 11 Samples		0–150 5 Samples	0–2000 7 Samples	0–400 7 Samples	80–140 4 Samples	0–400 10 Samples	0–250 7 Samples
**Volume**	1 µL	N/A	1.56 µL		N/A	Few mL ^#^	300 µL	Droplet	N/A	5 mL
**Contact- less**	Contact	Contact	Contactless		Contact	Contactless	Contactless	Contact	Contactless	Contactless
**Coefficient determination (R^2^)**	0.9997 N/A	0.995 0.9985	0.88471 N/A	0.97185 N/A	0.9955 0.9893	N/A 0.9726	N/A 0.9628	N/A 0.9999	N/A 0.998	0.9956 0.9927
**Human trial**	No	No	No	No	No	No	No	Yes	No	No
**Material under test (MUT)**	DI water/ Water-glucose	DI water/ Water-glucose	DI water/Water-glucose		DI water/ Water-glucose	DI water/ Water-glucose	DI water/ Water- glucose	Blood	DI Water/ ABPS	DI water/ Water-glucose
**Number of ports**	Double	Single	Single	Single	Single	Double	Double	Double	Double	Double
**Size (A) [mm^2^]**	7.74 × 7.74	13 × 7	20.5 × 9.2	5 × 5	40 × 45	37 × 18	73.3 × 30	85.7 × 85.7	49 × 45	25 × 15
Norm. size (A/λ_g_^2^) *	0.0578 ^#^	0.0370 ^#^	0.795 ^#^	0.100 ^#^	0.438 ^#^	0.820 ^#^	5.18 ^#^	18.27 ^#^	0.537 ^#^	0.1366
**Biosensor structure**	Single Asymmetric SRR	CSRR	CSRR (Reader)	DCC-SRR (Tag)	Hexagonal CSRR	CSRR	CSRR	SRR	DCC-SRR	Concentric Square SRR

^#^ Calculated from available data and figures in the article. * Normalized size is calculated as A/λ_g_^2^, where λ_g_ = c/*f*_0_√ε_r_ is the guided wavelength on the respective substrate.

## Data Availability

The original contributions presented in this study are included in the article. Further inquiries can be directed to the corresponding author.

## Abbreviations

The following abbreviations are used in this manuscript:

ABPS	Artificial Blood Plasma Solution
ASRR	Asymmetric Split Ring Resonator
BGL	Blood Glucose Level
BLE	Bluetooth Low Energy
CFRD	Cystic Fibrosis–Related Diabetes
CPW	Coplanar Waveguide
CSRR	Complementary Split-Ring Resonators
CST	Computer Simulation Technology
DCC	Double Concentric Circular
DI Water	Deionized Water
DM	Diabetes Mellitus
EM	Electromagnetic
EMI	Electromagnetic Interference
FCC	Federal Communications Commission
FR-4	Flame Retardant 4
GDM	Gestational Diabetes Mellitus
IDC	Interdigital Capacitors
ISM	Industrial-Scientific-Medical
LTE	Long Term Evolution
MUT	Material Under Test
N/A	Not Available
NIR	Near-Infrared
PDMS	Polydimethylsiloxane
PCB	Printed Circuit Board
RF	Radio Frequency
SD	Standard Deviation
SMBG	Self-Monitoring of Blood Glucose
SOL	Short-Open-Load
SRR	Split-Ring Resonator
T1D	Type 1 Diabetes
T2D	Type 2 Diabetes
UWB	Ultra-Wide Band
VNA	Vector Network Analyzer
Wi-Fi	Wireless-Fidelity
